# Comparison of the efficacy and characteristics of metallic foreign body extraction by incision surgery and x-ray guided forceps after body-surface projection positioning: A STROBE-compliant article

**DOI:** 10.1097/MD.0000000000012116

**Published:** 2018-08-21

**Authors:** Hexiang Qian, Xianju Qin, Guangfu Xing, Changwen Shi, Li Zhang

**Affiliations:** Department of Surgery, Shanghai Eighth People's Hospital, Shanghai, China.

**Keywords:** body surface projection positioning, metallic foreign body extraction, soft tissue, x-ray

## Abstract

A foreign body retained in soft tissue may give rise to infection and dysfunction, which may pose a potential threat to patient health. Our study is to compare the efficacy and characteristics of metallic foreign body (MFB) extraction from soft tissue by incision surgery and x-ray-guided forceps after body surface projection positioning.

This study enrolled 775 patients who underwent percutaneous MFB extraction between January 2011 and December 2016. A total of 257 cases underwent extraction by incision surgery and 518 cases underwent x-ray-guided forceps extraction after body surface projection positioning.

All patients were diagnosed by x-ray and the diagnostic accuracy rate was 100%. In the incision surgery group, MFB extraction was successful in 193 of 257 cases. All cases in the forceps extraction group were successful, and the success rate was significantly higher than that of the incision surgery group (100% vs.75.1%, *P* < .01). Sixty-four patients in the incision surgery group who failed treatment were subsequently treated with x-ray-guided forceps extraction and all MFBs were extracted. The symptoms in all patients were relieved, wound healing was good, and there were no major bleeding, incision infection, or other complications.

Compared with incision surgery, x-ray-guided foreign body forceps extraction after body surface projection positioning is a less invasive, safer, and more effective treatment for MFB extraction.

## 1 Introduction

A foreign body retained in soft tissue is caused by the penetration of a foreign body in the human body and may pose a potential threat to patient health.^[1,2]^ Conventional treatment involves a skin and soft tissue incision under local anesthesia to remove the foreign body. Briefly, the operator searches for the foreign body in the appropriate area after the tissues are incised and isolated. A foreign body can be touched and explored using the fingers even if it is in the deep tissues. However, the foreign body may not be touched when tissue swelling induced by local anesthesia is present. The foreign body can move with blunt separation or traction during surgery. If the incision is too small, the foreign body may be difficult to extract, and the approach often requires a larger incision; in addition, it is difficult to extract a foreign body which is small or cannot be touched on the body surface.^[3]^

There are 2 types of foreign bodies: metallic and non-metallic.^[4]^ The x-ray-impermeable characteristic of a metallic foreign body (MFB) is helpful for the diagnosis and precise localization of this type of foreign body.^[5]^ Injury caused by a foreign body generally results in a relatively small wound, but is often accompanied by deep soft tissue injury. Organs, blood vessels, and nerves can also be damaged.^[1]^ In some cases, the wound on the skin is small, but exploration using vascular forceps or the fingers may find injuries in deep tissues. Bleeding is usually not serious, but a hematoma can form in the wound channel. In this study, we analyzed our experience in the diagnosis and treatment of 775 patients with a retained MFB admitted to our hospital during the past 6 years.

## 2 Patients and methods

### 2.1 Patient population

A total of 775 patients (620 men and 155 women aged from 15 to 59 years with an average age of 39 years) with MFBs who were treated in the Surgical Emergency Department of our hospital between January 2011 and December 2016 were included in this study. This study was approved by the Ethics Committee of Shanghai Eighth People's Hospital and all patients provided written informed consent. The size and the distribution of different MFBs are shown in Tables 1 and 2.

**Table 1:**
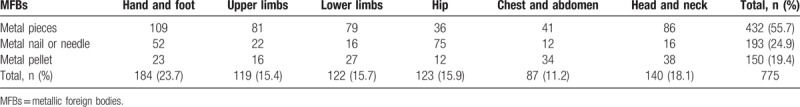
Distribution of MFBs in the body.

**Table 2:**
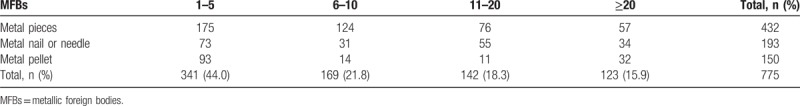
Maximum long diameter of the MFBs (millimeter).

### 2.2 Surgical approaches

We selected patients who had MFBs which were superficial, large, easily touched, distant from vital organs, and had a clear positional relationship with blood vessels, and performed incision foreign body extraction surgery. In patients with MFBs which were deep, small, difficult to touch, and close to vital organs or blood vessels, we performed x-ray-guided foreign-body forceps extraction after body surface projection positioning. MFB extraction surgery was performed as soon as possible after diagnosis.

**Figure 1:**
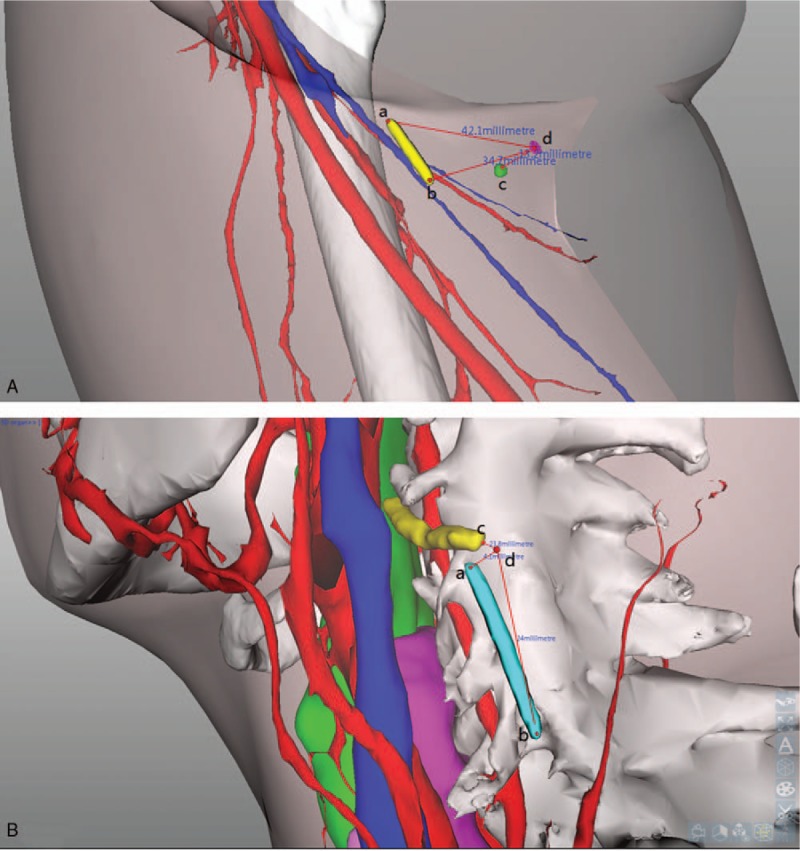
3D images. (A) 3D image of the body-surface projection of a spherical foreign body in the right thigh (yellow ab represents body-surface marker line; green c represents the foreign body; purple d represents the image which the foreign body projected to the body surface with the shortest distance). (B) 3D image of a needle-shaped foreign body near the cervical vertebral artery (blue ab represents body-surface marker line; yellow c represents the foreign body; d represents the surgical puncture point in the skin).

**Figure 2:**
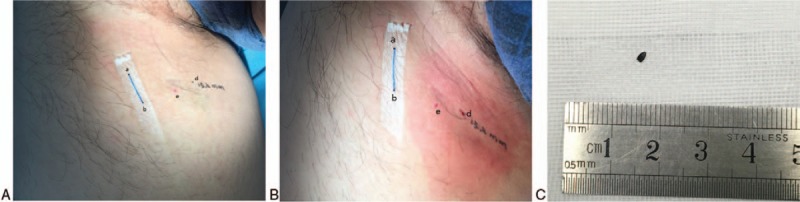
Surgical incision markers on the skin of the thigh. (A) Preoperative positioning markers in a patient with a thigh foreign body (ab represents body-surface marker line; d represents the site where the foreign body was projected in the body surface with the shortest distance; e represents the site where the foreign body penetrated the body). (B) Postoperative image of the patient with the thigh foreign body (d represents the incision). (C) Extracted foreign body.

**Figure 3:**
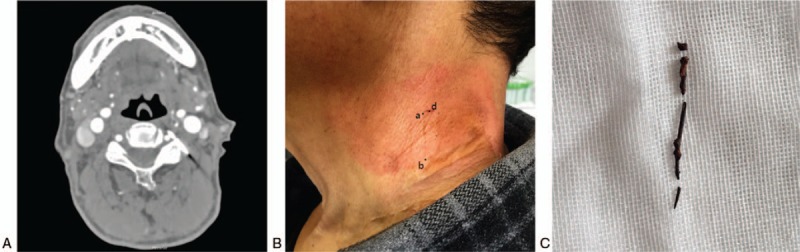
Surgical incision markers on the skin of the neck. (A) CT image of a needle-shaped foreign body near the cervical vertebral artery. (B) Postoperative image of the patient with the needle-shaped foreign body near the cervical vertebral artery (ab represents the location of the body-surface marker line; d represents the incision). (C) Extracted foreign body.

In the surgical group, incision surgery was performed to extract the MFB. If a foreign body was touched on the body surface, the site on the skin closest to the foreign body was marked. Local infiltration anesthesia or nerve block anesthesia was administered according to the location of the foreign body. Skin and subcutaneous tissues at the marked site were carefully incised and isolated until the foreign body was found and removed. If the foreign body was located in deeper tissues and could not be touched on the body surface, the direction of penetration of the foreign body was judged according to the wound on the skin as well as patient information combined with palpation and exploration using vascular forceps. Needle positioning was also used, and the operator used the needle to determine the location of the foreign body, by repeatedly pulling and pushing the needle in different directions. When the needle showed some resistance or the needle was heard to touch the foreign body, the position and direction of the needle were fixed and the incision was extended until the foreign body was accessed, exposed, and extracted.

x-ray-guided foreign-body forceps extraction was performed after body surface projection positioning. A positioning marker line was attached to the skin close to the foreign body. The surgical area was scanned using computed tomography (CT) and three-dimensional (3D) images were reconstructed (Fig. 1). The images displayed the skin, bones, shallow veins, blood vessels, and vital organs around the foreign body, and their position in relation to the foreign body. The size of the foreign body, the depth from the skin surface, and the position in relation to surrounding vital organs and the marker were measured. The 2 ends of the positioning marker line were set as A and B, and the target was C; the best puncturing point nearest the foreign body avoiding vital organs was set as D. The pathway for percutaneous puncturing was from D to C. The body surface projection of the foreign body was helpful for finding a pathway vertical to the skin surface with the shortest distance between the foreign body and the skin. The skin was punctured vertically along the marker of the body surface projection with the anesthesia needle and the foreign body was often touched by this method. Thus, selection of the puncturing point under x-ray during surgery was avoided. This reduced the radiation exposure time and avoided blood vessels, nerves, and scar tissues. Surgical incision was performed on the marked skin (Figs. 2 and 3).

**Figure 4:**
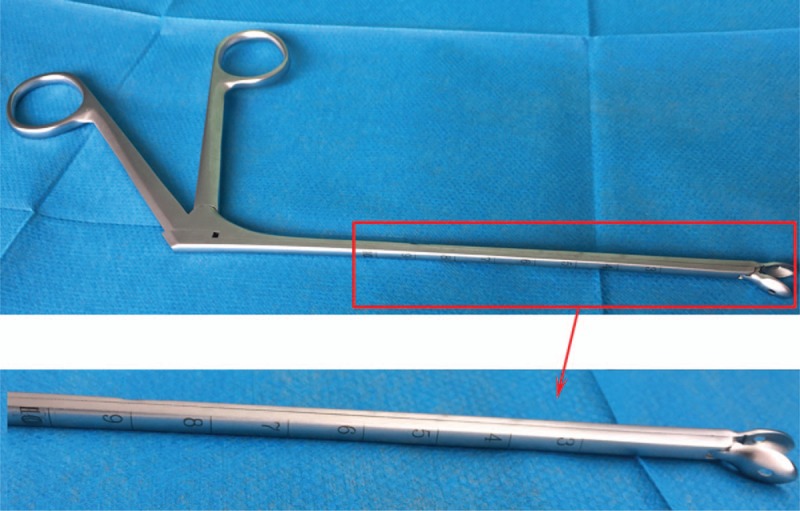
Foreign body forceps used in surgery.

When the position, size, and depth of the foreign body were known, foreign body forceps with an appropriate head and length were used (Fig. 4). The skin at the pre-marked site was incised under local anesthesia. Guided by x-ray, the foreign body forceps were then inserted. The top of the foreign body forceps is smooth and blunt, which avoids important vessels and nerves when in contact with soft tissues, thus minimizing injury to surrounding tissues. In addition, foreign body forceps have accurate calibration providing the operator with information on the exact depth of the forceps in the tissues. Foreign body forceps are generally equipped with a magnetic component which can attract MFBs. Following foreign body extraction into the interior wall of the foreign body forceps, the forceps are slowly retracted and the foreign body removed. This method has advantages including minimal trauma, less bleeding, convenience, and a high success rate. The surgical wound does not need sutured because incision surgery is not required, and postoperative complications can also be avoided.

### 2.3 Statistical analysis

Data were analyzed with SPSS (version 22.0; SPSS, Chicago, IL). Student *t* test was used to determine the statistical significance of differences between the groups. Differences with a *P* value <.05 were considered statistically significant.

## 3 Results

### 3.1 Success rate

A total of 775 patients were diagnosed by x-ray or CT and the diagnostic accuracy rate was 100%. The patients underwent either incision surgery to extract MFBs, or x-ray-guided foreign body forceps extraction after body-surface projection positioning. Of 257 patients (203 men and 54 women aged from 21 to 57 years with an average age of 34 years) who underwent incision surgery, foreign bodies were extracted in 193 cases with a 75.1% success rate, whereas 64 patients who failed incision surgery and another 518 relatively complicated cases (417 men and 101 women aged from 15 to 59 years with an average age of 41 years) underwent x-ray-guided foreign body forceps extraction. Foreign bodies were extracted in all 582 patients. There was a significant difference in the success rate between the 2 groups (75.1% vs.100%, *P* < .01). In the 64 patients who failed incision surgery, surgery was halted in 35 patients because of severe pain and in the other 29 patients because of longer operation time without removal of the MFBs. This did not occur in the forceps extraction group, and the difference between the 2 groups was significant (*P* < .01). Five patients in the incision surgery group developed numbness of the skin distal to the incision because of nerve injury. None of the patients in the forceps extraction group developed severe postoperative complications, and the incidence of complications was significantly different between the 2 groups (*P* < .01). Symptoms were relieved in all patients. At the 1-week outpatient follow-up visit, all wounds were healing well, with no major bleeding, incision infection, or other major complications.

### 3.2 Comparison of the two approaches

x-ray-guided forceps extraction had a high success rate, small incision, low incidence of complications, short surgery time, reduced use of anesthetics and less bleeding, and was superior to incision surgery (Table 3). Furthermore, x-ray-guided forceps extraction was a better choice for small MFBs retained in deep tissues.

**Table 3:**
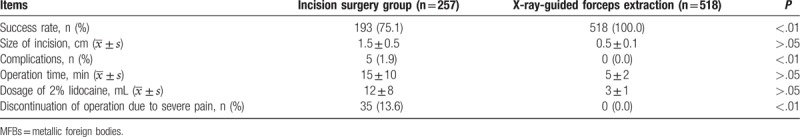
Comparison of incision surgery and x-ray-guided forceps extraction of MFBs.

## 4 Discussion

The key to extraction of foreign bodies is accurate positioning. x-ray, CT, and ultrasound are all able to display the position of a foreign body in soft tissue and its relationship with other organs or tissues.^[4,6,7]^ Ultrasound, particularly high-frequency ultrasound, has significant clinical value in positioning metallic and non-MFBs.^[8–10]^ Under x-ray guidance, foreign body forceps or small hemostatic forceps can be used to explore and extract the foreign body from the original wound; however, the wound channel may be distorted if the patient's position is changed. In addition, images shown on the screen are 2D images, which lack stereoscopic view^[11,12]^; these factors may increase the difficulty in surgery. Our approach was to set a marker on the body surface and the developing line in medical gauze was used as a positioning marker line. These materials are easily obtained. The images were clear with no metal artifacts. We performed CT scanning to identify the most appropriate surgical pathway, position the foreign body using the body-surface marker, and obtained body surface projection images by calculation. The choice of surgical pathway should take into account the shortest pathway and avoid surrounding organs, and if it is a fresh wound, the puncture point can also be located at the wound surface, and the foreign body may be extracted using forceps directly through the wound track. During CT examination, projection positioning, and surgery, the patient should maintain the same body position to reduce error. A needle-shaped foreign body can easily change its location with body movement; therefore, it is recommended that the lesion is immobilized after positioning and surgery is completed as quickly as possible. The target can be the foreign body, tissues or organs depending on the CT display and the images projected to the body surface can be calculated. This method has advantages including accurate positioning, availability of related materials, and intuitive images after body-surface projection marking.^[13]^ This method is also convenient for the operator.

If the MFB is in the vascular area, preoperative examination requires enhanced CT to clarify the location of the MFB and blood vessels. Iodinated contrast media (ICM) are commonly used in enhanced CT imaging. Adverse effects owing to ICM include the following 3 categories of symptoms^[14]^: mild symptoms include dizziness, headache, nausea, vomiting, sneezing, cough, conjunctival congestion, measles, and so on. Moderate symptoms include limb edema, hoarseness, and low blood pressure, and severe symptoms can cause shock and even death. Therefore, the iodine allergy test should be conducted before the administration of ICM, and first aid preparation for adverse effects also should be carried out. In our study, there were no allergic reactions or complications with ICM.

3D images can be reconstructed after CT scanning, which not only display the location of the foreign body and its relationship with the surrounding organs, but also facilitates optimization of the surgical pathway by accurately measuring the distance between the foreign body and incision using the marker line.^[15,16]^ Specially designed foreign body forceps can be used to extract foreign bodies under x-ray guidance. Foreign body forceps are equipped with precise calibration and a blunt spindle-shaped head, which prevents the puncture of blood vessels during the operation. Moreover, there is a magnetic patch in the head of the forceps to attract MFBs. This method does not require incision surgery and has advantages such as accurate positioning of the foreign body, short operation time, is a relatively simple device, low cost, less trauma and a high success rate, and is in line with the trend in minimally invasive treatment principles.^[17,18]^

If foreign-body forceps extraction cannot avoid vital organs, incision surgery in combination with x-ray-guided forceps extraction can be performed. In some situations, the foreign body can be extracted by thoracoscopy or laparoscopy combined with x-ray-guided forceps extraction. All of these techniques greatly expand the application of forceps extraction.

Lammers et al^[19]^ suggested that MFBs retained in the soft tissue do not pose a potential hazard to the surrounding organs, and if the patient has no symptoms, the foreign body may not require removal. However, some foreign bodies can move from the original location along blood vessels because of frequent muscle activity. A proportion of free foreign bodies were found in our study. The penetration of a foreign body can not only cause direct impact injury, contusion and laceration, pain, and swelling, but can also lead to local bleeding, infection and the formation of a hematoma or pseudoaneurysm.^[4,20–22]^ The long-term risks of a foreign body also include metal corrosion, foreign body granuloma, and even the induction of a tumor as well as secondary injuries if the foreign body moves.^[23,24]^ Therefore, retention of MFBs is detrimental to patients. Foreign bodies endangering vital organs must be extracted promptly. The remaining sharp MFBs must also be removed as soon as possible to avoid movement of these foreign bodies in soft tissues and organ injury.

The x-ray-impermeable characteristic of MFBs facilitates the diagnosis, positioning, and extraction of such foreign bodies. Non-MFBs have many similarities to MFBs. However, non-MFB cannot be displayed in x-ray images. Although there are reports that non-MFBs were extracted guided by molybdenum target x-ray or super high frequency ultrasound, the overall efficacy is inferior to that of MFBs. Most non-MFBs are extracted by incision surgery. It is helpful for the diagnosis and treatment of non-MFBs to understand the characteristics of incision surgery and forceps extraction of MFBs.

## Author contributions

**Conceptualization:** Hexiang Qian.

**Data curation:** Xianju Qin, Changwen Shi.

**Formal analysis:** Hexiang Qian.

**Investigation:** Hexiang Qian, Guangfu Xing.

**Methodology:** Hexiang Qian.

**Resources:** Hexiang Qian.

**Software:** Changwen Shi.

**Writing – original draft:** Guangfu Xing, Li Zhang.

**Writing – review & editing:** Xianju Qin.
